# Adverse pregnancy outcomes associated with antiretroviral therapy initiated before pregnancy and during pregnancy: a retrospective study in Hubei province, China

**DOI:** 10.3389/fmed.2023.1158962

**Published:** 2023-05-18

**Authors:** Yuting Tan, Songjie Wu, Yajun Yan, Shi Zou, Ling Feng, Wei Guo, Mengmeng Wu, Mingqi Luo, Ke Liang

**Affiliations:** ^1^Department of Infectious Diseases, Zhongnan Hospital of Wuhan University, Wuhan, China; ^2^Wuhan Research Center for Infectious Diseases and Cancer, Chinese Academy of Medical Sciences, Wuhan, China; ^3^Department of Nosocomial Infection Management, Zhongnan Hospital of Wuhan University, Wuhan, China; ^4^Department of Pathology, Zhongnan Hospital of Wuhan University, Wuhan, China; ^5^Department of Pathology, School of Basic Medical Sciences, Wuhan University, Wuhan, China; ^6^Hubei Engineering Center for Infectious Disease Prevention, Control and Treatment, Wuhan, China

**Keywords:** pregnancy outcome, HIV, antiretroviral therapy, lopinavir/ritonavir, nonnucleoside reverse transcriptase inhibitors

## Abstract

**Background:**

Antiretroviral therapy (ART) initiation before pregnancy was reported to have an increased risk of adverse pregnancy outcomes (APOs) than ART initiation during pregnancy. However, the risks of APOs associated with different ART regimens initiated before or during pregnancy remain unknown.

**Methods:**

Pregnant women living with HIV (PWLHIV) from Hubei Province, China, were retrospectively enrolled between January 1, 2004, and December 31, 2021. The trends of ART initiation time and application of different ART regimens were evaluated over time, separately. Using no ART exposure before and during pregnancy as control, the risks of APOs associated with protease inhibitor (PI) based regimens and non-nucleoside reverse transcriptase inhibitors (NNRTIs) based regimens initiated before pregnancy were analyzed; and the risks of APOs associated with PI-based regimens, NNRTIs based regimens and zidovudine (AZT) monotherapy initiated during pregnancy were analyzed. APOs, including low birthweight (LBW), stillbirth, preterm birth (PTB) and early miscarriage, were reviewed.

**Results:**

Among 781 PWLHIV including 1,010 pregnancies, 522 pregnancies (51.7%) were exposed to ART before or during pregnancy. Of them, the proportion of ART initiation before pregnancy per year increased from around 20% in the early period to more than 60% after 2019. Efavirenz (EFV)-nucleoside reverse transcriptase inhibitors (NRTIs) (32.2%), LPV/r-NRTIs (31.2%), and nevirapine (NVP)-NRTIs (27.4%) were the most commonly used regimens, and the proportion of LPV/r-NRTIs used per year has increased to around 50.0% in recent years. LPV/r-NRTIs was associated with higher risks of LBW whether initiated before pregnancy [adjusted OR (aOR) = 2.59, 95%CI 1.04–6.45, *p* = 0.041] or during pregnancy (aOR = 2.19, 95%CI 1.03–4.67, *p* = 0.041), compared with no exposure to ART before and during pregnancy. However, no matter initiated before or during pregnancy, LPV/r-NRTIs had no significantly increased risks of stillbirth, PTB and early miscarriage, and EFV /NVP-NRTIs and AZT monotherapy had no significantly increased risks of LBW, stillbirth, PTB and early miscarriage when compared with no exposure to ART before and during pregnancy.

**Conclusion:**

Our data suggests that LPV/r-NRTIs has been widely used among PWLHIV in recent years. However, the potential risk of LBW should be continuously monitored among PWLHIV whether LPV/r-NRTIs is initiated before or during pregnancy.

## Introduction

About 37.7 million people were reported to be living with human immunodeficiency virus (HIV) globally in 2020, and among them, 19.3 million were women of childbearing age (1). Facing the increasing proportion of HIV infection among pregnant women, antiretroviral therapy (ART) has effectively improved maternal health and prevented mother-to-child transmission (2, 3). However, ART-related risk of adverse pregnancy outcomes (APOs) has been deeply concerned in recent years.

Currently, the World Health Organization (WHO) recommends dolutegravir (DTG) combined with nucleoside reverse transcriptase inhibitors (NRTIs) as the first-line regimen, efavirenz (EFV) combined with NRTIs as the alternative first-line regimen, and protease inhibitor (PI) based ART including lopinavir/ritonavir (LPV/r) as an alternative regimen for pregnant women living with HIV (PWLHIV) (4). In China, LPV/r combined with NRTIs is recommended as the first-line regimen for PWLHIV (5). PI-based ART, especially LPV/r and non-nucleoside reverse transcriptase inhibitor (NNRTI), including EFV or nevirapine (NVP) based ART may be associated with the increased risks of APOs among PWLHIV, although research results remain controversial in different studies (6–9).

PWLHIV with ART initiation before pregnancy were reported to have higher risks of APOs, including preterm birth (PTB), small for gestational age (SGA) and low birth weight (LBW) than those with ART initiation during pregnancy (10–13). Further evidence showed that ART initiation before conception had an negative effect on the placental histopathology and pregnancy outcomes than ART initiation during pregnancy (14). Given that more evidence indicated that ART initiation before or during pregnancy affected pregnancy outcomes, it is necessary to evaluate the risks of APOs for different ART regimens initiated before and during pregnancy. However, very few studies have focused on assessing these outcomes in China. Our previous study found that ART exposure in the first trimester was a risk factor of APOs (15), but the differences in risk by different ART regimens and APO outcomes were not further analyzed.

As PI, especially LPV/r, and NNRTI-based regimens are widely used among PWLHIV in China, understanding the risk of different APO associated with PI-based ART and NNRTIs based ART initiated before and during pregnancy is vital for informing prevention of mother-to-child transmission programming. A meta-analysis revealed that maternal HIV infection naive to ART was associated with an increased risk of APOs, including PTB, SGA, LBW and stillbirth, in comparison to HIV-negative women (16). Here we extended our previous study (15) by analyzing the risk of different APO associated with PI-based ART and NNRTIs based ART initiated before and during pregnancy, in comparison to no ART exposure before and during pregnancy.

## Methods

### Study population

All pregnant women with confirmed HIV infection during the antenatal care period were retrospectively recruited from Center for Disease Control and Prevention (CDC) at all levels in Hubei Province, China, between January 1, 2004, and December 31, 2021. All those PWLHIV were managed and followed by the CDC and maternal and child health care hospitals in different districts. For women who had more than one pregnancy during the study period, each pregnancy was treated as a separate event. All pregnancies were followed up to 18 months postpartum. For pregnancies which were terminated, the follow-up was ended. For analyzing APOs risks for different ART regimens, pregnancies without ART exposure before and during pregnancy were considered as controls. Exclusion criteria for the APOs risk analyses of different ART regimens were as follows: (1) pregnancies with unknown pregnancy outcomes due to voluntary termination or ectopic pregnancy; (2) pregnancies lost to follow-up; (3) ART regimen was switched or stopped during pregnancy; (4) pregnancies exposed to other ART regimens; (5) pregnancies exposed to unknown ART regimens or unknown ART initiation time.

### Data collection

Study data between 2004 and 2021 were retrospectively collected from medical records of the CDC and maternal and child health care hospitals in different districts. The following data were extracted: maternal age at conception, gravidity, year of pregnancy, hepatitis B virus (HBV) infection status (confirmed by HBsAg test), hepatitis C virus (HCV) infection status (confirmed by HCV RNA test), date of HIV diagnosis, smoking during pregnancy, alcohol use during pregnancy, mode of HIV transmission, maternal CD4+ T lymphocyte count (CD4 count) at delivery, the timing of ART initiation (before or during pregnancy), ART regimens use before and during pregnancy, adverse pregnancy outcomes including low birthweight (LBW), stillbirth, preterm birth (PTB) and early miscarriage.

### Definition and grouping

The first, second, and third trimesters were defined as less than 14 weeks of gestation, 14 weeks to the end of 27 weeks of gestation, and 28 weeks of gestation to delivery, respectively. The total APOs included early miscarriage (noninduced pregnancy loss at <12 weeks of gestation), stillbirth (fetal death at ≥20 weeks of gestation), PTB (gestational age < 37 completed weeks at delivery), and LBW (birth weight < 2,500 g at delivery) (17, 18).

The APOs risks of different ART initiated before pregnancy and APOs risks of different ART initiated during pregnancy were analyzed, separately. For APOs risks analyses of different ART initiated before pregnancy, since no PWLHIV had been exposed to AZT monotherapy before pregnancy in our study, the groups of ART exposure were divided as follows: (1) PI-NRTIs; (2) NNRTI-NRTIs; (3) no ART exposure before and during pregnancy. For APOs risks analyses of different ART initiated during pregnancy, the groups of ART exposure were divided: (1) PI-NRTIs; (2) NNRTI-NRTIs; (3) no ART exposure before and during pregnancy; (4) AZT monotherapy.

### Statistical analysis

SPSS 21.0 and Graphpad Prism 5.0 were used for data statistics and plotting. Continuous variables were denoted as medians with the 25th to 75th interquartile range (IQR). Categorical variables were denoted as counts (%). Maternal age, gravidity, HBV or HCV co-infection, the HIV transmission mode, the timing of HIV diagnosis, CD4 count at delivery, smoking and alcohol intake, and year of pregnancy were included in the univariate Poisson regression analysis of the risk of APOs. Variables with *p* < 0.1 in the above univariate Poisson regression analysis were considered confounding variables to adjust the multivariate Poisson regression analysis of the total APO and different APO risks associated with different ART. Pregnancies without ART exposure before and during pregnancy were considered as a reference. *p* < 0.05 was considered statistically significant.

## Results

### Participants’ characteristics

Overall, 781 PWLHIV including 1,010 pregnancies, were recruited from Hubei Province, China, between January 1, 2004, and December 31, 2021. Of them, 488 exposed no ART, 522 (51.9%) pregnancies were exposed to ART before pregnancy or during pregnancy. The percentage of ART initiated before pregnancy (173/522, 33.1%) was higher than ART initiated during pregnancy at first (128/522, 24.5%), second (123/522, 23.5%) and third (98/522, 18.7%) trimester. From 2004 to 2021, LPV/r was the only PI used, and EFV and NVP were the only NNRTI used. EFV-NRTIs (168/522, 32.2%), LPV/r-NRTIs (163/522, 31.2%) and NVP-NRTIs (143/522, 27.4%) were the most commonly used ART regimens.

**Figure 1:**
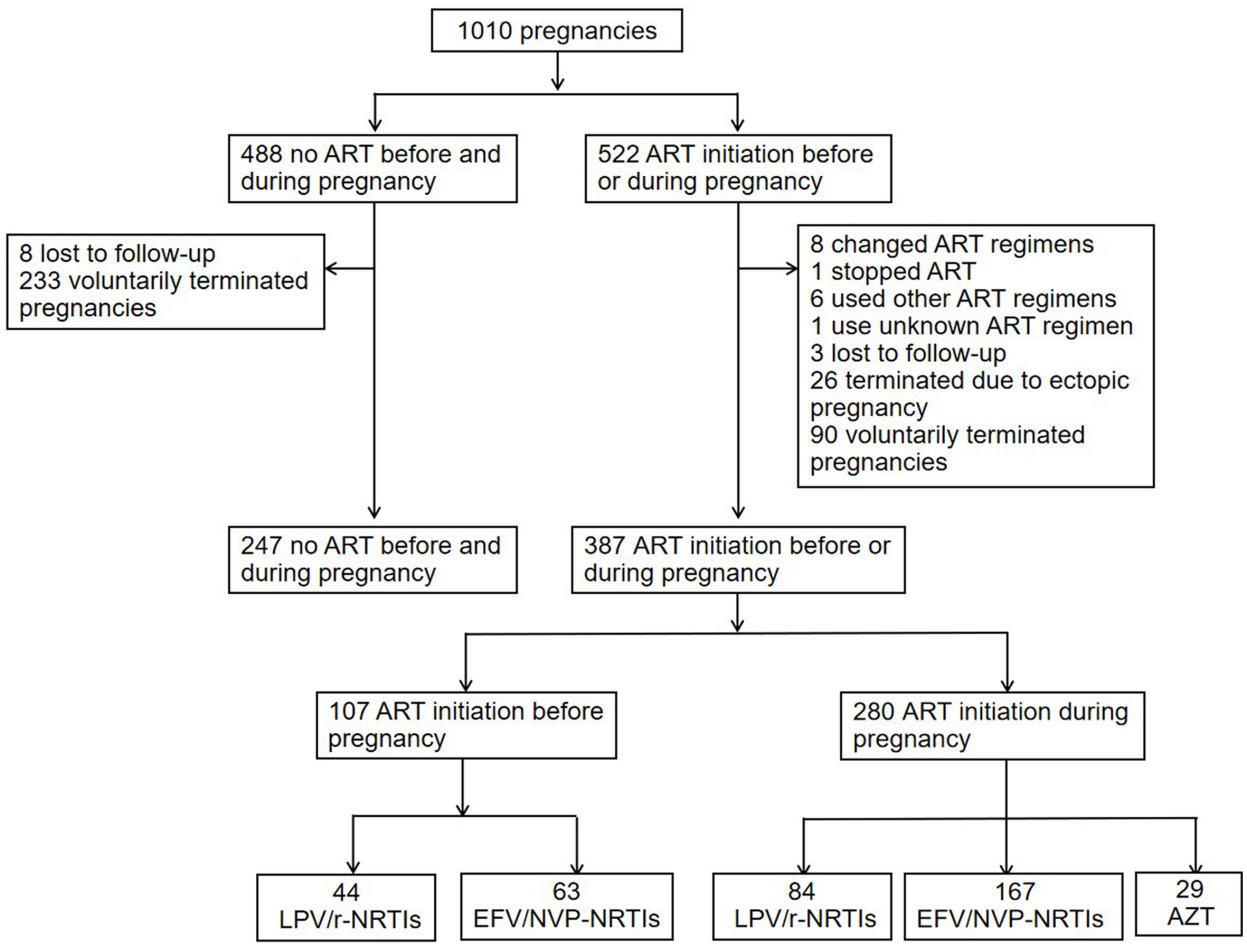
Flowchart of pregnancies included in the APOs analysis of different ART regimens. ART, antiretroviral therapy; PI, protease inhibitor; LPV/r, lopinavir/ritonavir; NRTI, nucleoside reverse transcriptase inhibitor; NNRTI, non-nucleoside reverse transcriptase inhibitor; EFV, efavirenz; NVP, nevirapine; AZT, zidovudine; APOs, adverse pregnancy outcomes.

Of 488 pregnancies with no ART exposure, 8 were lost to follow-up, and 233 voluntarily terminated their pregnancies. Therefore, 247 pregnancies with no ART exposure included in the analysis of APOs risks associated with different ART regimens. Of 522 pregnancies exposed to ART before pregnancy or during pregnancy, 8 changed ART regimens, one stopped ART, 6 used other ART regimens, one use unknown ART regimen, 3 were lost to follow-up, 26 terminated pregnancies due to ectopic pregnancy, and 90 voluntarily terminated their pregnancies. Finally 387 pregnancies (107 exposed to ART before pregnancy, 280 exposed to ART during pregnancy) included in the analysis of APOs risks associated with different ART regimens. For APOs risks analyses of different ART regimens initiated before pregnancy, 44 pregnancies were exposed to LPV/r-NRTIs, and 63 were exposed to NNRTI (EFV or NVP, EFV/NVP)-NRTIs. For APOs risks analyses of different ART regimens initiated during pregnancy, 84 were exposed to LPV/r-NRTIs, 167 were exposed to EFV/NVP-NRTIs, and 29 were exposed to AZT monotherapy. 247 pregnancies were unexposed to ART before and during pregnancy (study controls). The flowchart of the study population enrolled was shown in Figure 1. The characteristics and proportions of pregnancies exposed to different ART regimens before pregnancy and pregnancies exposed to different ART regimens during pregnancy were shown in Table 1. Among 247 pregnancies with no ART exposure, 34 (13.7%) infants with HIV infetion were observed. Among 167 pregnancies with EFV/NVP-NRTIs initiation during pregnancy, one (0.6%) infant with HIV infection was found.

**Table 1:** Characteristics of pregnancies exposed to different ART regimens before pregnancy or during pregnancy.

	No ART (*n* = 247)	ART initiation before pregnancy		ART initiation during pregnancy		
		LPV/r-NRTIs (*n* = 44)	EFV/NVP-NRTIs (*n* = 63)	LPV/r-NRTIs (*n* = 84)	EFV/NVP-NRTIs (*n* = 167)	AZT monotherapy (*n* = 29)
**Maternal age [years, *n* (%)]**						
<25	80 (32.4)	6 (13.6)	9 (14.3)	24 (28.6)	38 (22.7)	18 (62.1)
25–35	140 (56.7)	33 (75.0)	42 (66.7)	53 (63.1)	109 (65.3)	10 (34.5)
≥35	27 (10.9)	5 (11.4)	12 (19.0)	7 (8.3)	20 (12.0)	1 (3.3)
**Year of pregnancy, *n* (%)**						
2004–2008	95 (38.5)	0 (0.0)	2 (3.2)	0 (0.0)	17 (10.2)	11 (37.9)
2009–2011	60 (24.3)	1 (2.3)	7 (11.1)	5 (5.9)	36 (21.6)	17 (58.6)
2012–2015	53 (21.4)	6 (13.6)	9 (14.3)	25 (29.7)	61 (36.5)	1 (3.4)
2016–2021	39 (15.8)	37 (84.1)	45 (71.4)	54 (64.3)	53 (31.7)	0 (0.0)
HBV or HCV co-infection, *n* (%)	15 (6.1)	4 (9.1)	3 (4.7)	10 (11.9)	12 (7.2)	1 (3.4)
Sexual transmission of HIV^§^, *n* (%)	226 (91.5)	43 (97.7)	53 (84.1)	81 (96.4)	158 (94.6)	24 (82.7)
HIV diagnosis before pregnancy, *n* (%)	22 (8.9)	44 (100.0)	63 (100.0)	34 (40.5)	73 (43.7)	5 (17.2)
CD4 count at delivery [cells/ul, median (IQR)]	325 (198–465)	580 (500–689)	500 (384–652)	489 (369–605)	396 (294–506)	481 (360–568)
**Maternal CD4 count at delivery [cells/ul, *n* (%)]**						
<250	85 (34.4)	1 (2.3)	3 (4.7)	7 (8.3)	21 (12.6)	0 (0.0)
250–500	100 (40.5)	9 (20.4)	22 (34.9)	40 (47.6)	96 (57.5)	15 (51.7)
≥500	51 (20.6)	31 (70.5)	30 (47.6)	36 (42.8)	47 (28.1)	14 (48.3)
Missing	11 (4.5)	3 (6.8)	8 (12.7)	1 (1.2)	3 (1.8)	0 (0.0)
Smoking during pregnancy, *n* (%)	20 (8.1)	3 (6.8)	3 (4.7)	6 (7.1)	11 (6.6)	0 (0.0)
Alcohol intake during pregnancy, *n* (%)	13 (5.3)	3 (6.8)	4 (6.3)	5 (5.9)	9 (5.4)	0 (0.0)
**Gravidity, *n* (%)**						
1	235 (95.1)	4 (9.1)	4 (6.3)	81 (96.4)	153 (91.6)	26 (89.7)
≥2	12 (4.9)	40 (90.9)	59 (93.7)	3 (3.6)	14 (8.4)	3 (10.3)
Infant HIV infection, *n* (%)	34 (13.7)	0 (0.0)	0 (0.0)	0 (0.0)	1 (0.6)	0 (0.0)

§ Refers to mode of maternal HIV infection. ART, antiretroviral therapy; LPV/r, lopinavir/ritonavir; NRTI, nucleoside reverse transcriptase inhibitor; EFV, efavirenz; NVP, nevirapine; HBV, hepatitis B virus; HCV, hepatitis C virus; CD4 count, CD4 T lymphocyte count; AZT, zidovudine.

### Trends of ART use over time

**Figure 2:**
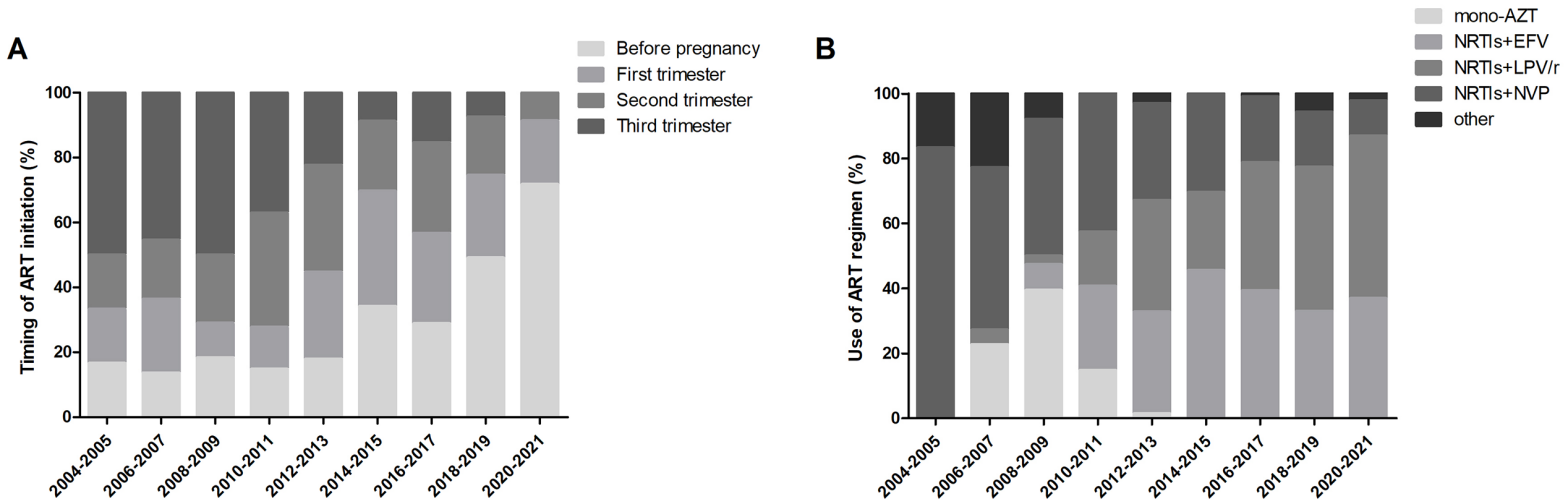
Trends of ART use over time. **(A)** Timing of ART initiation among 522 pregnancies over time. **(B)** ART regimens used among 522 pregnancies over time. AZT, zidovudine; NRTI, nucleoside reverse transcriptase inhibitor; EFV, efavirenz; LPV/r, lopinavir/ritonavir; NVP, nevirapine.

Of the 522 pregnancies with ART initiation before or during pregnancy, the timing of ART initiation varied over time (shown in Figure 2A). In 2004, none was on ART before and during pregnancy. From 2005, the proportion of ART initiated before pregnancy per year increased from around 20% in the early period to >60% after 2019. The trend of ART initiated in the third trimester per year declined over time, from around 50% in the early period to less than 10% after 2018. Of the 522 pregnancies receiving ART, different ART regimens used over time were analyzed. As shown in Figure 2B, LPV/r-NRTIs and EFV-NRTIs were the most frequently used regimens after 2011. The proportion of LPV/r-NRTIs use per year increased from <10% in the early period to around 50.0%, while the proportion of NVP-NRTIs use declined from >40% in the early period to around 10% in recent years. The proportion of EFV-NRTIs use per year remained stable at more than 30%.

### APOs risks associated with different ART regimens initiated before pregnancy

As shown in Table 2, LPV/r-NRTIs initiation before pregnancy was associated with a significantly increased risk of LBW (adjusted OR (aOR) = 2.59, 95%CI 1.04–6.45, *p* = 0.041) but with no significant increased risks of total APOs, stillbirth, PTB and early miscarriage compared with no ART exposure before and during pregnancy., Also, there was no significant increase in the risks of total APOs, LBW, stillbirth, PTB, and early miscarriage associated with EFV/NVP-NRTIs initiated before pregnancy compared with no ART exposure before and during pregnancy.

**Table 2:** Multivariable analysis of APOs risks associated with different ART regimens initiated before pregnancy or during pregnancy.

	No ART (*n* = 247)	ART initiation before pregnancy		ART initiation during pregnancy		
		LPV/r-NRTIs (*n* = 44)	EFV/NVP-NRTIs (*n* = 63)	LPV/r-NRTIs (*n* = 84)	EFV/NVP-NRTIs (*n* = 167)	AZT monotherapy (*n* = 29)
Total APOs, *n*	20	9	13	16	22	1
adjusted OR (95%CI)	1 [Ref]	6.13 (0.70–53.24)	5.69 (0.61–52.44)	1.47 (0.77–2.83)	1.09 (0.64–1.85)	0.55 (0.07–4.40)
*p* value		0.100	0.125	0.241	0.759	0.575
LBW, *n*	13	6	5	12	15	0
adjusted OR (95%CI)	1 [Ref]	2.59 (1.04–6.45)	1.58 (0.59–4.23)	2.19 (1.03–4.67)	1.38 (0.69–2.74)	–
*p* value		0.041	0.360	0.041	0.357	–
Stillbirth, *n*	4	1	3	1	4	0
adjusted OR (95%CI)	1 [Ref]	1.40 (0.16–12.26)	2.94 (0.67–12.80)	0.73 (0.08–6.48)	1.48 (0.37–5.83)	–
*p* value		0.759	0.151	0.782	0.576	–
PTB, *n*	6	3	4	8	4	1
adjusted OR (95%CI)	1 [Ref]	2.52 (0.84–7.54)	1.92 (0.76–4.86)	2.37 (0.86–6.54)	0.59 (0.20–1.72)	1.28 (0.13–12.56)
*p* value		0.098	0.165	0.095	0.339	0.830
Early miscarriage, *n*	2	1	4	2	3	0
adjusted OR (95%CI)	1 [Ref]	0.70 (0.02–26.02)	1.62 (0.10–27.37)	1.87 (0.18–19.16)	1.18 (0.20–6.95)	–
*p* value		0.850	0.736	0.595	0.855	–

APOs, adverse pregnancy outcomes; ART, antiretroviral therapy; LPV/r, lopinavir/ritonavir; NRTI, nucleoside reverse transcriptase inhibitor; EFV, efavirenz; NVP, nevirapine; AZT, zidovudine; OR, odds ratio; LBW, low birthweight; PTB, preterm birth.

### APOs risks of different ART regimens initiated during pregnancy

As shown in Table 2, LPV/r-NRTIs initiation during pregnancy was associated with a significantly increased risk of LBW (aOR = 2.19, 95%CI 1.03–4.67, *p* = 0.041) compared with no ART exposure before and during pregnancy. No significant increase in risks of total APOs, stillbirth, PTB and early miscarriage were found to be associated with LPV/r-NRTIs initiated during pregnancy. Similarly, no significantly increased risks of total APOs, LBW, stillbirth, PTB and early miscarriage were found to be associated with EFV/NVP-NRTIs and AZT monotherapy initiated during pregnancy.

## Discussion

Global concerns about the increasing APOs among PWLHIV has been rising in recent years. Previous studies found that PWLHIV exposed to ART before conception were significantly more likely to have APOs than all other PWLHIV or those exposed to ART post-conception (10, 19). Hence, further understanding the APOs risks associated with different ART regimens initiated before and initiated during pregnancy is relevant for ART regimens selection and development of APO monitoring strategies for PWLHIV. This study supplements the limited data on the risks of various APO associated with LPV/r-based and NNRTI-based regimens according to ART initiation time.

In our study, the proportion of ART initiation before pregnancy increased per year, rising to more than 60% after 2019. This observation was in line with the findings of other studies in the US, Botswana, and France (20, 21). Since 2015, the WHO has recommended early ART initiation in HIV-positive women regardless of CD4 count (22). Therefore, the use of ART before pregnancy has rapidly increased since most women living with HIV are of childbearing potential (1). Consistent with the results of other studies in Europe (6, 23), we observed that LPV/r-based regimens and NNRTI, especially EFV-based regimens, were the commonly used ART regimen among PWLHIV. The proportion of LPV/r use has been increasing in recent years, per our study findings. This finding is expected and can be attributed to the recommendation of an experts consensus in China that LPV/r combined with NRTIs as the first-line regimen and EFV or DTG or NVP combined with NRTIs are the alternative regimens for PWLHIV (5).

The Promoting Maternal and Infant Survival Everywhere (PROMISE) large-scale multisite randomized open-label trial suggested that both LPV/r-AZT-lamivudine (3TC) and LPV/r-tenofovir disoproxil fumarate (TDF)-emtricitabine (FTC) significantly increased the incidence of LBW than AZT monotherapy (24). Our study further showed that LPV/r-NRTIs initiation before pregnancy and LPV/r-NRTIs initiation during pregnancy were related to a significantly higher risk of LBW, compared with no ART exposure before and during pregnancy. Similarly, findings from a meta-analysis on the association between adverse perinatal outcomes and antenatal ART regimens showed that antenatal LPV/r-NRTIs use had the highest risk of LBW (8). Increased risk of LBW caused by LPV/r may be related to increased estradiol levels (25). One study found that HIV-positive women exposed to LPV/r-based regimens were likely to have an increased estradiol level than HIV-positive women without ART exposure (26). Moreover, studies on mouse models revealed that maternal high estradiol levels might contribute to the increased risk of LBW (27, 28). And, decreased progesterone levels caused by PI use may also contribute to LBW (29). Therefore, the increased risk of LBW should be monitored in HIV-positive pregnant women on ART whether LPV/r is initiated before conception or during pregnancy. Since LPV/r is still recommended as the first-line treatment for PWLHIV in China (5).

In the guidelines from the United States, United Kingdom and Europe, atazanavir/ritonavir (ATV/r) and darunavir/ritonavir (DRV/r), but not LPV/r, have been recommended to be the preferred PIs for PWLHIV since the concern that LPV/r may be associated with increased risk of PTB (30–32). In our study, PTB incidence was higher among pregnancies with LPV/r-NRTIs initiation either before pregnancy or during pregnancy than in those without ART exposure before and during pregnancy. However, further multivariate analysis revealed no significant increased risk of PTB associated with LPV/r-NRTIs initiated before and during pregnancy. PI, especially LPV/r based regimens, have been reported to be associated with an increased risk of PTB in comparison to AZT monotherapy (8, 24) or NNRTIs based regimens (7) or non-PI based regimens (33), although the related data still remains controversial (6, 34–36). A multi-center observational study across eight European countries found an increased risk of PTB among PWLHIV with LPV/r-based regimens initiation before pregnancy but no increased risk of PTB associated with LPV/r-based regimens initiated during pregnancy (23). Their study findings suggest that the timing of LPV/r initiation may affect the risk of PTB. Moreover, PWLHIV receiving booster PIs were more likely to have PTB than those receiving unboosted PIs (37, 38). Variations in sample size, heterogeneity of the control group, timing of LPV/r initiation, and booster LPV/r use may have contributed to the contradicting results in reported by relevant research studies.

Our study assessed the risks of APOs associated with EFV-based and NVP-based combination regimens, namely NNRTI-based regimens. We found that NNRTI (EFV/NVP)-NRTIs initiation either before pregnancy or during pregnancy was not associated with a significant increase risks of LBW, PTB, or stillbirth compared with no exposure to ART. A large retrospective observational study in Botswana revealed that EFV-TDF-FTC initiated before pregnancy had the lowest risk of adverse birth outcomes, including stillbirth, PTB, SGA, and neonatal death, than LPV/r combined with NRTIs (7). A systematic review have summarized the risks of PTB associated with different ART regimens, finding that EFV-based regimens exhibited null effects or were protective against PTB when compared with all other regimens (39). Compared to HIV-negative women, HIV-positive women receiving EFV-TDF-3TC had a similar risk of PTB, LBW, and SGA (40). All these findings suggest that EFV-NRTIs may be relatively safe among PWLHIV. Currently inconsistent data on the comparison of APOs risks between EFV-based regimens and NVP-based regimens have been reported. Data from previous studies revealed that EFV-based regimens initiation preconception and post-conception was not associated with any significant differences in the risks of stillbirth, LBW, miscarriage, PTB, and SGA from NVP-based ART (41, 42). However, there were also studies showing that NVP-based regimens had increased risks of PTB and LBW than EFV-based regimens (39). More research is needed in the future to confirm this observation since our study finding was limited by sample size of EFV/NVP-NRTIs regimen users.

We also found no significant increase in the risks of LBW, PTB, or stillbirth associated with AZT monotherapy initiated during pregnancy compared to no ART exposure before and during pregnancy. On the contrary, a meta-analysis including 409,781 pregnant women indicated that PWLHIV with AZT monotherapy were associated with a decreased risk of PTB and LBW, compared with ART-naive PWLHIV (8). We also found no significant increase in the risk of early miscarriage associated with LPV/r-NRTIs and EFV/NVP-NRTIs initiated before pregnancy or initiated during pregnancy compared to no ART exposure before and during pregnancy. Few studies have reported on the risks of miscarriage associated with ART use. A prospective study involving 2,113 pregnant women in sub-Saharan Africa found no evidence of an association between ART use in PWLHIV and pregnancy loss. However, the risk of pregnancy loss associated with the timing of ART initiation and different ART regimens was not analyzed (43). Therefore, more research studies exploring the association between early miscarriage risk and different ART regimens among PWLHIV are needed.

Some limitations exist in our study. First, the sample size for the APOs analysis was limited and may not be representative of the larger population. However, we still found a significant increase in the risk of LBW associated with LPV/r-NRTIS initiated before pregnancy and LPV/r-NRTIS initiated during pregnancy. This preliminary finding could inform ART treatment monitoring in pregnant women and spur further research on subject. Second, some PWLHIV terminated pregnancy voluntarily and were not included in the APO analysis, which may have led to some bias in the study results. Therefore, our finding should be interpreted with caution. Third, consistent with previous studies (7, 34, 44), we did not take HIV viral load as a variable in the analysis of APOs risks. Due to the limited availability of HIV viral load tests in the early stages, data on HIV viral load in most pregnant women are missing. More large sample, prospective studies are needed to explore the APOs risks associated with different ART regimens according to the timing of ART initiation in the future.

## Conclusion

Our study showed that LPV/r-NRTIs have been widely used among PWLHIV in recent years. Whether LPV/r-NRTIs were initiated before pregnancy or during pregnancy, the risk of LBW increased in pregnant women who used this regimens. Therefore, continued monitoring of LBW risks is necessary among PWLHIV who initiated LPV/r-based regimens before and during pregnancy.

## Data availability statement

The original contributions presented in the study are included in the article/supplementary material, further inquiries can be directed to the corresponding author/s.

## Ethics statement

The studies involving human participants were reviewed and approved by the institutional ethics committee of Zhongnan Hospital of Wuhan University (20210035). The patients/participants provided their written informed consent to participate in this study.

## Author contributions

KL and ML conceived and designed this investigation. YT, SW, YY, SZ, LF, WG, and MW collected the original data. YT and SW analyzed the data. YT and KL contributed to the writing of the paper. All authors contributed to the article and approved the submitted version.

## Funding

This work was supported by Medical Science and Technology Innovation Platform Support Project of Zhongnan Hospital, Wuhan University (PTXM2020008), Science and Technology Innovation Cultivation Fund of Zhongnan Hospital, Wuhan University (cxpy2017043). Medical Science Advancement Program (Basic Medical Sciences) of Wuhan University (TFJC2018004).

## Conflict of interest

The authors declare that the research was conducted in the absence of any commercial or financial relationships that could be construed as a potential conflict of interest.

## Publisher’s note

All claims expressed in this article are solely those of the authors and do not necessarily represent those of their affiliated organizations, or those of the publisher, the editors and the reviewers. Any product that may be evaluated in this article, or claim that may be made by its manufacturer, is not guaranteed or endorsed by the publisher.
